# Shifts in Estimated Preferred Directions During Simulated BMI Experiments With No Adaptation

**DOI:** 10.3389/fnsys.2021.677688

**Published:** 2021-07-19

**Authors:** Miri Benyamini, Miriam Zacksenhouse

**Affiliations:** ^1^Brain-Computer Interfaces for Rehabilitation Laboratory, Faculty of Mechanical Engineering, Technion-Israel Institute of Technology, Haifa, Israel; ^2^Technion Autonomous Systems Program, Haifa, Israel

**Keywords:** brain-machine interfaces, BMI filter, preferred direction, shifts in preferred direction, neural modulations, neural encoding

## Abstract

Experiments with brain-machine interfaces (BMIs) reveal that the estimated preferred direction (EPD) of cortical motor units may shift following the transition to brain control. However, the cause of those shifts, and in particular, whether they imply neural adaptation, is an open issue. Here we address this question in simulations and theoretical analysis. Simulations are based on the assumption that the brain implements optimal state estimation and feedback control and that cortical motor neurons encode the estimated state and control vector. Our simulations successfully reproduce apparent shifts in EPDs observed in BMI experiments with different BMI filters, including linear, Kalman and re-calibrated Kalman filters, even with no neural adaptation. Theoretical analysis identifies the conditions for reducing those shifts. We demonstrate that simulations that better satisfy those conditions result in smaller shifts in EPDs. We conclude that the observed shifts in EPDs may result from experimental conditions, and in particular correlated velocities or tuning weights, even with no adaptation. Under the above assumptions, we show that if neurons are tuned differently to the estimated velocity, estimated position and control signal, the EPD with respect to actual velocity may not capture the real PD in which the neuron encodes the estimated velocity. Our investigation provides theoretical and simulation tools for better understanding shifts in EPD and BMI experiments.

## 1. Introduction

Firing rates of cortical motor neurons represent a diversity of motor, sensory, and cognitive signals, and most notably the direction and speed of movement (Georgopoulos et al., 1986; Georgopoulos, 2000; Johnson et al., 2001; Paz et al., 2003). In particular, center-out reaching experiments indicate that the firing rates of single cortical motor neurons are broadly “tuned” to the direction of movement. Changes in firing rates with the direction of movement are well described by a cosine function of the angle between movement direction and a neuron-specific direction, dubbed the preferred direction (PD). Detailed investigations suggest that the activity of directionally tuned cortical motor neurons is also modulated by the speed of movement (Moran and Schwartz, 1999). While the activity of PMd neurons are modulated mainly by the direction and amplitude of the movement (Messier and Kalaska, 2000; Hendrix et al., 2009), the activity of M1 neurons has been shown to correlate also with the applied forces (Ashe, 1997; Todorov, 2000).

During experiments with brain machine interfaces (BMIs), the estimated PDs (EPDs) of some neurons seem to shift after switching from manual control to brain control (Lebedev et al., 2005; Fan et al., 2014). The observed shifts in EPDs are usually assumed to reflect some process of adaptation, where the term “adaptation” describe any change in the actual tuning properties of the units, internal model or control strategy, including adaptation to changes in context (Green and Kalaska, 2011; Chase et al., 2012; Fan et al., 2014; Orsborn et al., 2014). Here we investigate whether shifts in EPDs may be observed after switching to brain control or changing BMI filters, even without any adaptation. Our hypothesis is that such shifts may occur without any adaptation, just due to the effect of imperfect BMI filters.

The investigation is conducted using the framework of optimal state estimation and feedback control (OFC) as a model for motor control (Todorov, 2005; Shadmehr and Krakauer, 2008). Optimal state estimation is achieved by integrating proprioceptive and visual measurements with prior state estimation from an internal model. Optimal gains are determined from a relevant cost function that penalizes for both control effort and inaccuracies in final position.

We have previously developed this framework to explain observed changes in neural modulations following the transition to brain control (Benyamini and Zacksenhouse, 2015). Optimal state estimation and control were conducted in the state-space, and neural activity encoded the estimated state and control signal. Spike counts were generated as realizations of doubly stochastic Poisson processes with linear tuning weights. The BMI filter was trained to decode the movement velocity from the neural activity.

Here we use this framework to investigate potential causes for the observed shifts in EPDs following the transition to brain control or to new BMI filters. In particular, we address the following research questions:

- Can the observed shifts in EPDs between different phases of BMI experiments occur even without any adaptation, i.e., without any change in the actual tuning properties of the recorded units or the internal model?
- Does the EPD in pole control agree well with the real PD of the unit, implied by the assigned tuning weights?
- Is the shift in EPD evident already in open-loop BMI, i.e., is there a shift between the EPD of the neural activity with respect to hand velocity and the EPD of the neural activity with respect to the velocity predicted (but not executed) by the BMI filter.
- Does the type of BMI filter affect the distribution of shifts in EPDs?
- Under what conditions, if any, are the EPDs expected to remain unaffected by the transition to brain control?

Due to inaccuracies in the BMI filter, movements generated in brain control differ from the intended movements, and thus may evoke adaptation. In the context of OFC models, adaptation may involve changes in the internal model, the estimation gains or the control gains (Green and Kalaska, 2011). In the context of neural encoding, adaptation may involve shifts in neural tuning properties, or functional change in network operation (Green and Kalaska, 2011; Chase et al., 2012; Fan et al., 2014; Orsborn et al., 2014). While adaptation may occur, here we investigate whether the observed shifts in EPD, following the transition to brain control, may occur even with no adaptation.

## 2. Materials and Methods

We investigate shifts in EPDs using both simulations and theoretical analysis. Simulation results are compared to experimental results reported in the literature from two groups: (1) Nicolelis' Lab (Carmena et al., 2003; Lebedev et al., 2005), and (2) Shenoy's Lab (Fan et al., 2014). The data recorded in Nicolelis Lab was further analyzed here to compute the histograms and estimate the distributions of PD shifts.

BMI experiments in Carmena et al. (2003) and Lebedev et al. (2005) involved movements to randomly placed targets, and used a linear BMI filter. The BMI experiments in Fan et al. (2014) included center-out reaching movements and used different types of Kalman filters. For consistency, we focus on simulations of the BMI experiments in Carmena et al. (2003) and Lebedev et al. (2005), with movements to randomly placed targets so the effects of the different BMI filters can be evaluated. Nevertheless, for completeness, we also present results from simulations of center-out movements. The BMI experiments are briefly described first, followed by the description of the analysis and modeling methods.

### 2.1. Experimental Methods

The BMI experiments in Carmena et al. (2003) and Lebedev et al. (2005) included three stages: pole control (PC), brain control with hand movements (BC-WHM) and brain control without hand movements (BC-WO-HM). During pole control the monkey controlled the position of a cursor on a computer screen by moving a hand-held pole. Neural activity was recorded from multiple brain areas, but mostly from the primary motor area (56 M1 units) and the dorsal pre-motor area (55 PMd units). Spike counts were binned into 100 ms bins and a linear filter was trained to predict the velocity from the current and previous 9 bins. Training was performed on data recorded during the last 10 min of pole control and held fixed during brain control. Initially, the monkey continued to move the hand even after the transition to brain control (BC-WHM), but eventually the monkey stopped moving the hand (BC-WO-HM). For more details, see Carmena et al. (2003).

The BMI experiments in Fan et al. (2014) included four stages: hand control and brain control with three types of Kalman filters. Data collected during hand control was used to build a Kalman filter (KF). Brain control with KF was referred to as KF online control. Data recorded during KF online control was used to re-calibrate the initial KF either directly, resulting in a re-calibrated KF (Re-KF), or after modifications that take into account the intention, resulting in a re-calibrated feedback intention trained Kalman filter (ReFIT-KF). Brain control with those filters was referred to as Re-KF online control and ReFIT-KF online control, respectively. Neural activity was recorded from M1 and PMd areas, and PD shifts were reported for 75–85 units in one Monkey and 30–45 units in the other Monkey.

To facilitate comparison with the respective literature, we use the terminology in Carmena et al. (2003) and Lebedev et al. (2005) to describe results derived from their experiments and from simulations of BMI experiments with linear filters. The terminology in Fan et al. (2014) is used to describe their results and the simulations of BMI experiments with Kalman filters.

### 2.2. Tuning Curves

Tuning curves and PDs of cortical neurons were initially estimated from spike trains recorded during center-out reaching movements by fitting mean spike counts per direction with a cosine function (Georgopoulos et al., 1986; Fan et al., 2014). These computations can be extended to estimate tuning curves and PDs during general reaching movements, including movement to randomly placed targets (Lebedev et al., 2005; Zacksenhouse and Nemets, 2008). This is usually performed by regressing the binned spike counts on the binned velocity. During general movements, the direction of movement may vary with time, so the neural activity in each bin may depend on the velocity at different bins. While tuning weights can be computed with respect to multiple bins simultaneously (Zacksenhouse and Nemets, 2008), here we follow (Lebedev et al., 2005) and apply linear regression with respect to individual bins:

(1) $$\begin{array}{r} N_{i}\left(t+\tau \right)=a_{i}\left(\tau \right)V_{x}\left(t\right)+b_{i}\left(\tau \right)V_{y}\left(t\right)+\epsilon_{i}\left(t,\tau \right) \end{array}$$

where *N*_*i*_ is the zero-mean neural activity of neuron *i*, τ is the lag, *a*_*i*_(τ) and *b*_*i*_(τ) are the regression coefficients, and ϵ_*i*_(*t*, τ) is the residual error (whose mean square value is minimized).

Thus, the estimated PD of neuron *i* at lag τ is defined by:

(2) $$\begin{array}{r} EPD_{i}\left(\tau \right)=arctan\left(\frac{b_{i}\left(\tau \right)}{a_{i}\left(\tau \right)}\right) \end{array}$$

The neural activity is usually regressed on cursor velocity (Equation 1). During PC, cursor velocity follows hand velocity, while during BC cursor velocity follows the velocity predicted by the BMI decoder. In order to assess the effect of the BMI filter on EPD shifts, we regressed the neural activity in pole control not only on the hand (pole) velocity, but also on the velocity predicted by the BMI filter. The latter is referred to as EPD in open-loop BMI.

### 2.3. Modeling BMI Experiments

**Figure 1:**
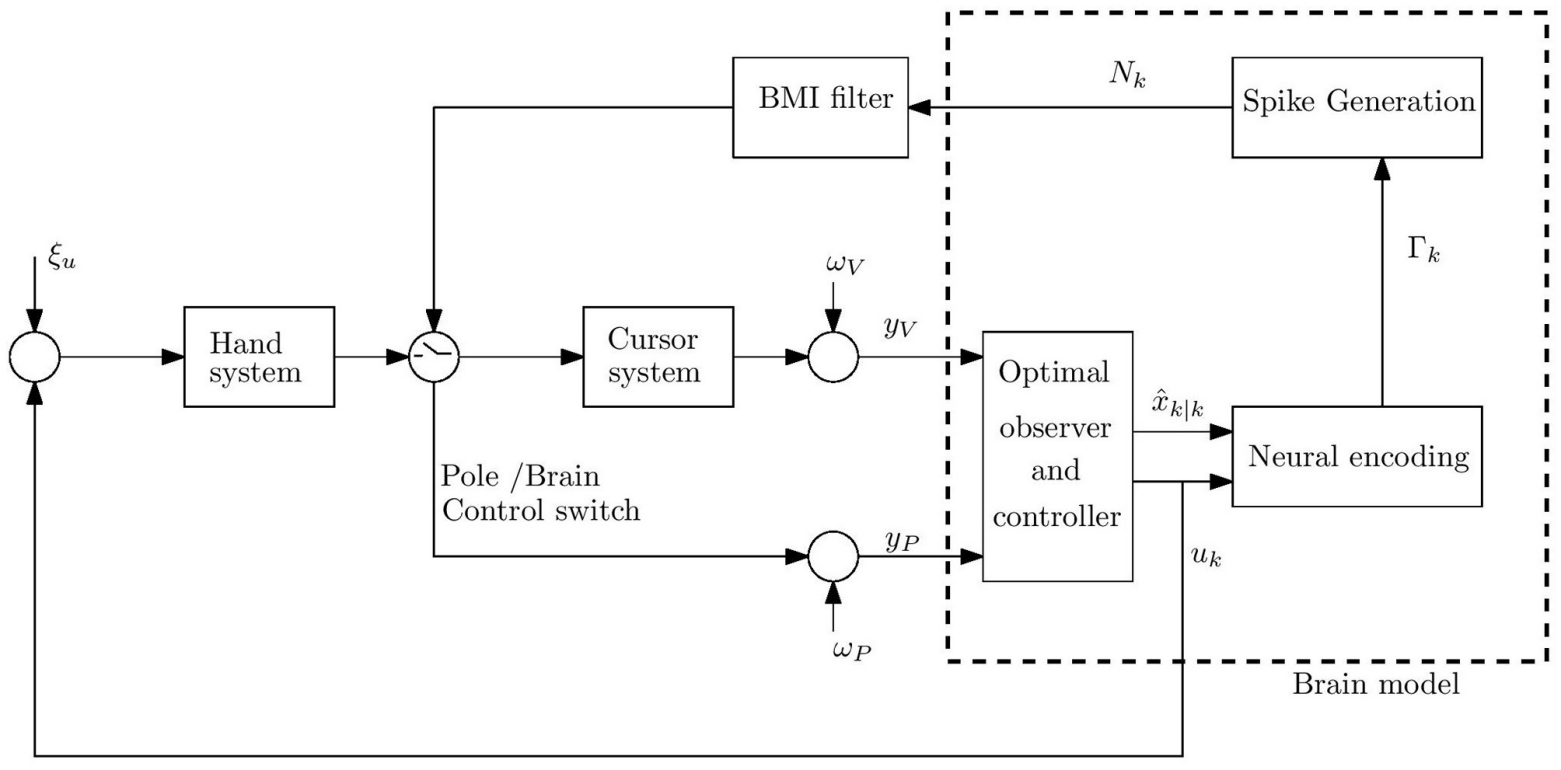
Schematic model of movement control during BMI experiments under the hypothesis that the brain implements optimal observer and controller. The neurons are assumed to encode the estimated state ${\hat{x}}_{k|k}$ and control signal, *u*_*k*_, in time step *k*. The cumulative bin rate Γ_*k*_ is a linear combination of the encoded signals (including the estimated speed and the magnitude of the control signal). The spike-count *N*_*k*_ is generated as a doubly stochastic Poisson process (DSPP) given Γ_*k*_. The control signal is corrupted by hand process noise ξ_*u*_. The brain model receives noisy proprioceptive *y*_*P*_ and visual *y*_*V*_ measurements from the hand and cursor, corrupted by proprioceptive and visual measurement noise, ω_*P*_ and ω_*V*_, respectively.

The model of BMI experiments is depicted in Figure 1 and briefly described here (for more details see Benyamini and Zacksenhouse, 2015). Following current computational motor control theories, the brain was assumed to implement optimal state estimation and feedback control (Wolpert et al., 1995; Todorov and Jordan, 2002; Todorov, 2005; Shadmehr and Krakauer, 2008). For simplicity, computations were performed in the state space, rather than their neural representations. BMI experiments were modeled by simulating the population of recorded units used by the BMI filter.

Thus, the brain model included three parts: (A) Observer that implements optimal state estimation by integrating sensory feedback (visual, *y*_*V*_, and proprioceptive, *y*_*P*_) with internal model predictions. The observer generates the estimated state, ${\hat{x}}_{k|k}$ at time step *k*. (B) Optimal controller with gains that minimize a standard cost function involving control effort and deviations from the target. The controller generates the control signal *u*_*k*_ from the estimated state. (C) Neural activity generator that generates spike counts *N*_*k*_ as realizations of doubly stochastic Poisson processes (Snyder, 1975; Zacksenhouse et al., 2007), given the cumulative bin rate Γ_*k*_, which encodes the relevant signals using linear multi-variable tuning weights, as further detailed below. Spike counts were generated in bins of 100 ms.

Two populations of 25 units each were simulated: (1) units that encode just the estimated state (2-dimensional vectors of estimated position and estimated velocity, and speed), and (2) units that also encode the 2-dimensional optimal control vector and its magnitude. Based on the evidence in the literature (Georgopoulos et al., 1982; Ashe and Georgopoulos, 1994; Ashe, 1997; Messier and Kalaska, 2000; Hendrix et al., 2009), we expect the behavior of simulated neurons in those two populations to be similar to the behavior of recorded PMd and M1 units, respectively, and hence refer to them as PMd-like and M1-like neurons. The 1:1 ratio between the number of M1-like and PMd-like units was based on a similar ratio (56:55) between the number of recorded M1 and PMd units in Carmena et al. (2003). The total number of units was selected in Benyamini and Zacksenhouse (2015) to achieve the reported BMI performance, as quantified by the coefficient of correlation between predicted and actual velocity in open-loop BMI.

We previously showed (Benyamini and Zacksenhouse, 2015) that the correlation between the velocity and neural activity peaked at −200 ms for M1 neurons and at 0 ms for PMd neurons, and that the correlation at −100 ms was close to the peak correlation for both populations. Thus, we focus on EPD shifts at −100 ms, but for completion report also EPD shifts at 0 ms and −200 ms.

In simulations with the ReFIT-KF, both populations also encoded movement intention in the form of a 2-dimensional target vector. Thus, each unit encoded up to four 2-dimensional vectors and can be characterized by up to four real PDs, one for each of those vectors (real PDs are defined in section 3, Equation 4).

The hand was modeled as a point mass driven by an over-damped second order muscle model that responds to the control signal from the brain (Todorov, 2005). A friction term was added to model the friction of the hand held pole as in Benyamini and Zacksenhouse (2015).

The optimal controller was designed to minimize a cost function that penalizes for deviations from the target at the desired reaching time and during subsequent 150 ms holding interval. Desired reaching times were uniformly distributed between 1.7*and*2.2 s during PC and 2.4−6 s in BC. The duration of the resulting simulated movements were comparable to those reported in Carmena et al. (2003), but about 4 times slower than those in Fan et al. (2014).

As in the BMI experiments (Carmena et al., 2003; Fan et al., 2014), the coefficients of the BMI filter were determined from simulated training data, i.e., velocity and neural activity during 10 min of simulated pole control. The BMI filter predicted the velocity based on the neural activity in the current and previous 9 bins. The resulting BMI filter was then used to predict the velocity and, by proper integration, the position of the cursor during simulated brain control. The BMI filter was also used to predict the velocity in PC in order to compute EPDs in open-loop BMI, as detailed at the end of section 2.2. PDs in PC and in open-loop BMI were computed from testing data, i.e., another section of 10 min that was not used to determine the BMI filter.

Kalman filters were determined as in Fan et al. (2014), with the same constraints on the elements of the transition matrix. In simulations with Re-KF and ReFIT-KF, the Kalman filter was re-calibrated during 20 min of BC with the initial KF. PDs were estimated from 20 min of simulated data in each stage.

### 2.4. Statistical Analysis of PD Shifts

Statistically significant shifts in EPDs were determined using bootstrap analysis, similar to the one conducted in Fan et al. (2014). Specifically, firing rates and velocities were re-sampled with replacement 1,000 times per stage, and 1,000 EPDs were computed for each stage and neuron. As mentioned before, here we compute the EPDs from movements in random directions using linear regression (Equation 1), while in Fan et al. (2014) EPDs were computed by fitting a cosine function to mean spike counts in 8 center-out directions. Thus, our bootstrap differs from the one in Fan et al. (2014) in re-sampling the data from all the directions rather than keeping the same number of samples per direction.

Following Fan et al. (2014), the distribution of shifts in EPDs between two specific stages was derived by computing the difference between 1,000 randomly sampled pairs of EPDs, one from each distribution. To assess statistical significance, “noisy” shifts were computed from zero-mean distributions of the EPDs in each stage (generated by subtracting the mean of the distribution). *T*-test was conducted for each neuron to evaluate if the shifts in EPDs between two stages were significantly larger than the noisy shifts (*p* < 0.05).

## 3. Theoretical Analysis of EPD Shifts

The model presented in section 2.3 and Figure 1 assumes that the neural activity encodes the estimated state (position and velocity) and control signal. Theoretical analysis relates (1) the EPD in PC to the real PD (RPD) with which the unit encodes the estimated velocity, and (2) the EPD in open-loop BMI to the EPD in PC. Since we focus on PC, for which the internal model is well adapted, the estimated state is assumed to be the same as the real state. Thus, theoretical analysis is based on the assumption that the neural activity encodes the real velocity, *V*, and other signals that are either correlated (e.g., control signal) or uncorrelated (e.g., speed) with the velocity (denoted by *C* & *U*, respectively). Specifically, the mean subtracted spike counts $N\in {\mathbb{R}}^{N_{n}\times T_{n}}$ of *N*_*n*_ neurons in *T*_*n*_ bins is assumed to be related linearly to the mean subtracted $V=\left[V_{x}\;V_{y}\right]\in {\mathbb{R}}^{2\times T_{n}}$, $C\in {\mathbb{R}}^{d_{C}\times T_{n}}$ and $U\in {\mathbb{R}}^{d_{U}\times T_{n}}$, by:

(3) $$\begin{array}{r} N=W_{V}V+W_{C}C+W_{U}U+\epsilon \end{array}$$

where *d*_*C*_ and *d*_*U*_ are the dimensions of the correlated and uncorrelated signals, respectively, $W_{V}\in {\mathbb{R}}^{N_{n}\times 2},\;W_{C}\in {\mathbb{R}}^{N_{n}\times d_{C}},\;W_{U}\in {\mathbb{R}}^{N_{n}\times d_{U}}$ are the tuning weights of *V*, *C* and *U*, respectively, and ϵ is the neural noise, which is assumed to be uncorrelated with any of the other signals.

The ratio between the *x* and *y* components of the tuning weights by which a specific unit encodes a specific signal defines the corresponding RPD. In particular, the RPD of neuron *i* with respect to velocity is:

(4) $$\begin{array}{r} RPD_{i}=arctan\left(\frac{W_{V_{x},i}}{W_{V_{y},i}}\right) \end{array}$$

To facilitate theoretical analysis, it is further assumed that the correlated signals can be expressed as *C* = *R*_*V*_*V* + *R*_*Z*_*Z* where *Z* is uncorrelated with *V*, so:

(5) $$\begin{array}{r} N=\left(W_{V}+W_{C}R_{V}\right)V+S \end{array}$$

where $S=W_{C}R_{Z}Z+W_{U}U+\epsilon \in {\mathbb{R}}^{N_{n}\times T_{n}}$ is uncorrelated with *V*.

The velocity tuning weights $\alpha =\left[a\;b\right]\in {\mathbb{R}}^{N_{n}\times 2}$ can be estimated by linear regression:

(6) $$\begin{array}{r} \alpha =NV^{+}=NV^{T}{\left(VV^{T}\right)}^{-1}\in {\mathbb{R}}^{N_{n}\times 2} \end{array}$$

The estimated tuning weights in PC, α_*PC*_, and in open-loop BMI, α_*BMI*_, are computed by regressing the neural activity in PC on either the hand velocity or the velocity predicted by the BMI filter, respectively.

First, we relate the tuning weights estimated in PC, α_*PC*_, to the real tuning weights, *W*_*V*_.

**Proposition 1A**: Assuming the neural activity can be modeled as in Equation 5, the tuning weights estimated from pole control, α_*PC*_, are given by:

(7) $$\begin{array}{r} \alpha_{PC}\approx W_{V}+W_{C}R_{V} \end{array}$$

**Proof of Proposition 1A:** Since *S* is uncorrelated with *V*, *SV*^*T*^ ≈ 0. Thus, Equation (7) can be derived directly by inserting Equation (5) in Equation (6):

(8) $$\begin{array}{r} \alpha_{PC}=\left(\left(W_{V}+W_{C}R_{V}\right)V+S\right)V^{T}{\left(VV^{T}\right)}^{-1}\approx \left(W_{V}+W_{c}R_{V}\right). \end{array}$$

Next we relate the tuning weights estimated in open-loop BMI, α_*BMI*_, to tuning weights estimated in PC, α_*PC*_. Proposition 1B expresses this relationship for the simple case when the BMI filter depends only on the current binned spike counts. Proposition 1C extends this to the general case when the BMI filter depends on the spike counts in the recent *L* bins.

**Proposition 1B**: Assuming the neural activity can be modeled as in Equation 5, and the BMI filter is based only on the current binned neural activity then the tuning weights in open-loop BMI, α_*BMI*_, are given by:

(9) $$\begin{array}{r} \alpha_{BMI}\approx \alpha_{PC}+\Sigma_{S}\alpha_{PC}{\left(\Sigma_{V}\Sigma_{\alpha_{PC}}\right)}^{-1}/N_{n} \end{array}$$

Where $\Sigma_{S}=SS^{T}/T_{n}\in {\mathbb{R}}^{N_{n}\times N_{n}},\Sigma_{V}=VV^{T}/T_{n}\in {\mathbb{R}}^{2\times 2}$ and $\Sigma_{\alpha_{PC}}=\alpha_{PC}^{T}\alpha_{PC}/N_{n}\in {\mathbb{R}}^{2\times 2}$ are the co-variance matrices of *S, V* and α_*PC*_, respectively.

**Proof of Proposition 1B:** In the simple case, the predicted velocity $\hat{V}\in {\mathbb{R}}^{2\times T_{n}}$ is a linear function of the zero mean neural activity $N\in {\mathbb{R}}^{N_{n}\times T_{n}}$ : $\hat{V}=W_{BMI}N$, Where $W_{BMI}\in {\mathbb{R}}^{2\times N_{n}}$ is the BMI decoder weights. The weights of the decoder are determined by linear regression of the zero-mean velocity *V*_*t*_ on the zero-mean neural activity *N*_*t*_ recorded for training:

(10) $$\begin{array}{r} W_{BMI}=V_{t}N_{t}^{+}=V_{t}N_{t}^{T}{\left\{N_{t}N_{t}^{T}\right\}}^{-1}=\frac{1}{T_{n}}V_{t}N_{t}^{T}\Sigma_{N}^{-1} \end{array}$$

Where $\Sigma_{N}=\frac{1}{T_{n}}N_{t}N_{t}^{T}$ is the co-variance matrix of the neural activity estimated from training data. As detailed in Supplementary Material, α_*PC*_*W*_*BMI*_ = *I*_*N*_*n*_×*N*_*n*__, and Equation (9) follows.

**Proposition 1C**: Assuming the neural activity can be modeled as in Equation 5, and that the BMI filter is based on the binned spike counts in the recent *L* bins of bin-width *B*_*W*_, the tuning weights in open-loop BMI, α_*BMI*_(*j*), in lag τ = *jB*_*W*_, are given by:

(11) $$\begin{array}{r} \alpha_{BMI}\left(j\right)=\alpha_{PC}\Sigma_{V}\left(\left|j-i\right|\right)\Sigma_{V}^{-1}\left(i\right)+\Sigma_{S}\left(\left|j-i\right|\right)\alpha_{PC}{\left(\Sigma_{V}\left(i\right)\Sigma_{\alpha_{PC}}\right)}^{-1}/N_{n} \end{array}$$

where $\Sigma_{S}\left(j\right)=S_{k}S_{k-j}^{T}/T_{n}$ and $\Sigma_{V}\left(j\right)=V_{k}V_{k-j}^{T}/T_{n}$ are the co-variance matrices of *S* and *V*, respectively, at lag τ = *jB*_*W*_, and $\Sigma_{\alpha_{PC}}=\alpha_{PC}^{T}\alpha_{PC}/N_{n}$ is the co-variance matrix of α_*PC*_.

**Proof of Proposition 1C:** Similar to the proof of Proposition 1B as detailed in Supplementary Material.

**Proposition 2**: Under the conditions of Proposition 1, if the co-variance matrices in Equation 11, Σ_*S*_(*j*) and Σ_*V*_(*j*) for *j* ≤ *L* and Σ_α_*PC*__, are scalar matrices (i.e., are proportional to the identity matrix), then the tuning weights in open-loop BMI at each lag are proportional to the tuning weights in pole control: α_*BMI*_(*k*) ∝ α_*PC*_.

Proof: Directly from Equation 11.

**Corollary**: Proposition 2 implies that under the indicated conditions, EPDs in PC are the same as EPDs in open-loop BMI (see Equation 2). However, the BMI experiments and the population of recorded units have to be well structured in order to satisfy those conditions:

- Σ_*V*_(*j*), *j* ≤ *L* are scalar matrices if *V*_*x*_ and *V*_*y*_ in different bins are uncorrelated and have the same co-variance.
- Σ_α_*PC*__ is a scalar matrix if during pole control, the two components of the tuning weights of all the units, α_*PC*_ = [*a*_*PC*_ *b*_*PC*_], are uncorrelated and have the same variance.
- Σ_*S*_(*j*), *j* ≤ *L* are scalar matrices if all the components of *Z* and *U*, which are included in *S*, are uncorrelated across different bins and have the same co-variance. Furthermore, the corresponding tuning weights should be ortho-normal, a condition that cannot be satisfied when the number of neurons is larger than the number of signals.

**Proposition 3**: Under the conditions of Proposition 1A, if *W*_*C*_ ∝ *W*_*V*_ and *R*_*V*_ is a scalar matrix, then α_*PC*_ ∝ *W*_*V*_.

Proof: directly from Equation 7.

**Corollary**: Proposition 3 implies that under the indicated conditions, EPDs in pole control are the same as RPDs (see Equations 2, 4). Again, the BMI experiments and the population of recorded units have to be well structured in order to satisfy those conditions:

- *W*_*C*_ ∝ *W*_*V*_ is satisfied if all the signals that are correlated with the velocity are encoded with the same RPD as the velocity.
- *R*_*V*_ is scalar matrix if all the signals that are correlated with the velocity are proportional to the velocity.

Those conditions are not under experimental control, and are not expected to be satisfied in regular experiments.

## 4. Results

### 4.1. PDs in BMI Experiments With Linear Filters

**Figure 2:**
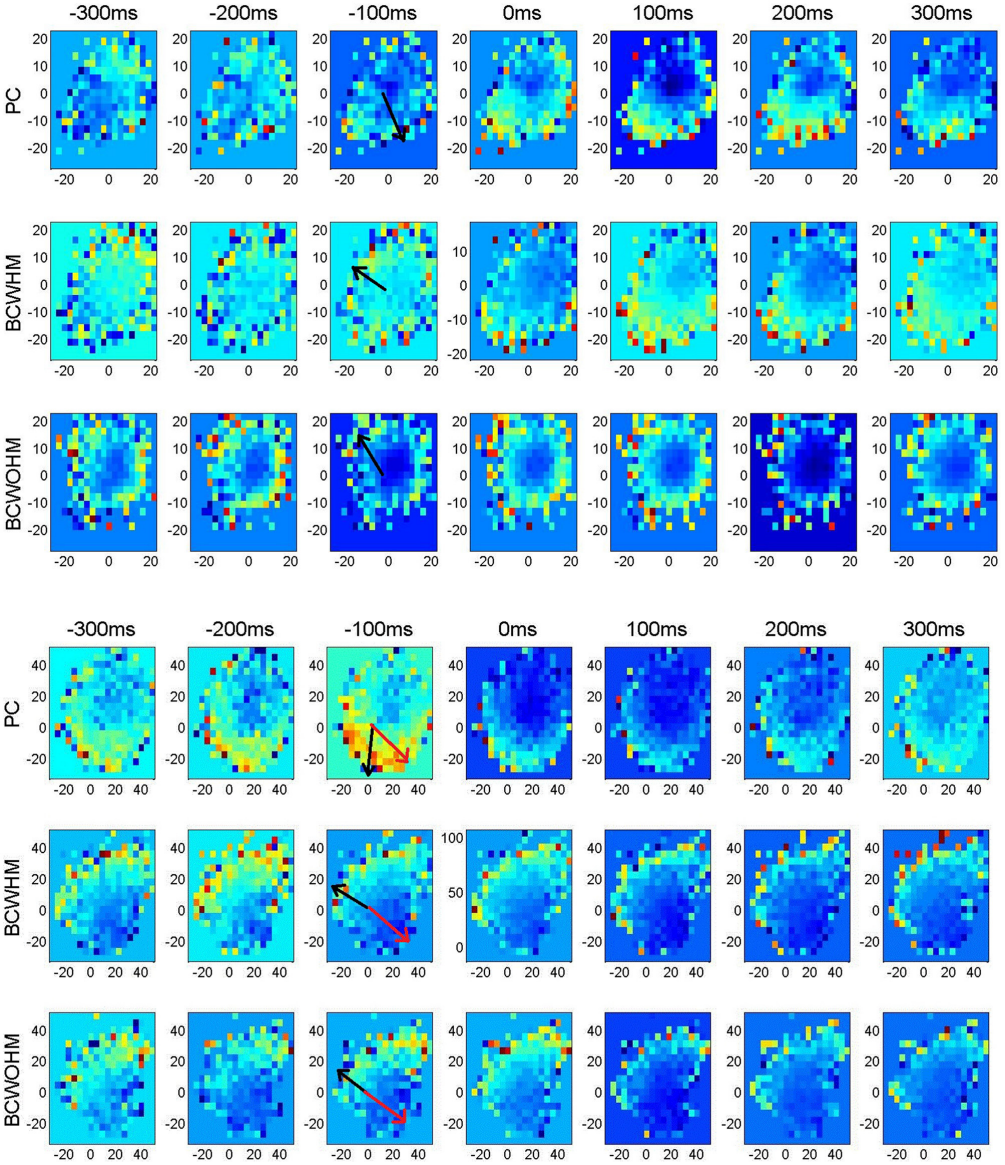
Velocity tuning of a recorded M1 unit (upper panels) and a simulated M1-like unit (lower panels) in different lags and stages demonstrating shifts in estimated PDs after switching from pole control (PC, top) to brain control with hand movements (BC-WHM, middle) or without hand movements (BC-WO-HM, bottom). Color plots of the mean spike-count (in bins of 100 ms) as a function of *V*_*x*_ (*x*-axis) and *V*_*y*_ (*y*-axis) in cm/s. Different color code for each panel. Estimated PDs at −100 ms are marked by black arrows, while actual PDs in simulations are marked by red arrows.

Figure 2 depicts representative velocity tuning of a recorded M1 unit (upper panels) and a simulated M1-like unit (lower panels). Velocity tuning is represented in 2-dimensional color plots where the color indicates mean spike-count in 100 ms bins as a function of *V*_*x*_ and *V*_*y*_. Velocity tuning for different stages of the experiment (PC, BC-WHM and BC-WO-HM) and lags (from −300 ms to +300 ms) are shown in different rows and columns, respectively. It is apparent that both the real and simulated units encode the speed (the magnitude of the velocity) and the direction of the velocity, since the mean firing rate changes with both the radius and the angle. The directions of the EPD at −100 ms (computed using Equation 2) are marked by black arrows.

The upper panels in Figure 2 demonstrate a case in which the EPD at −100 ms rotated by about 130° after switching from PC (EPD = 283.°, with respect to *x* axis) to BC-WO-HM (EPD = 151.4°). The lower panels in Figure 2 demonstrate that a similar rotation can also be observed in a simulated neuron. Here, the EPD at −100 ms rotated by about 115° from pole control (EPD = 267.8°) to BC-WHM (EPD = 153.7°). Furthermore, the EPD differed from the RPD, which was 328.2° as marked by red arrows, even in PC but especially in BC.

**Figure 3:**
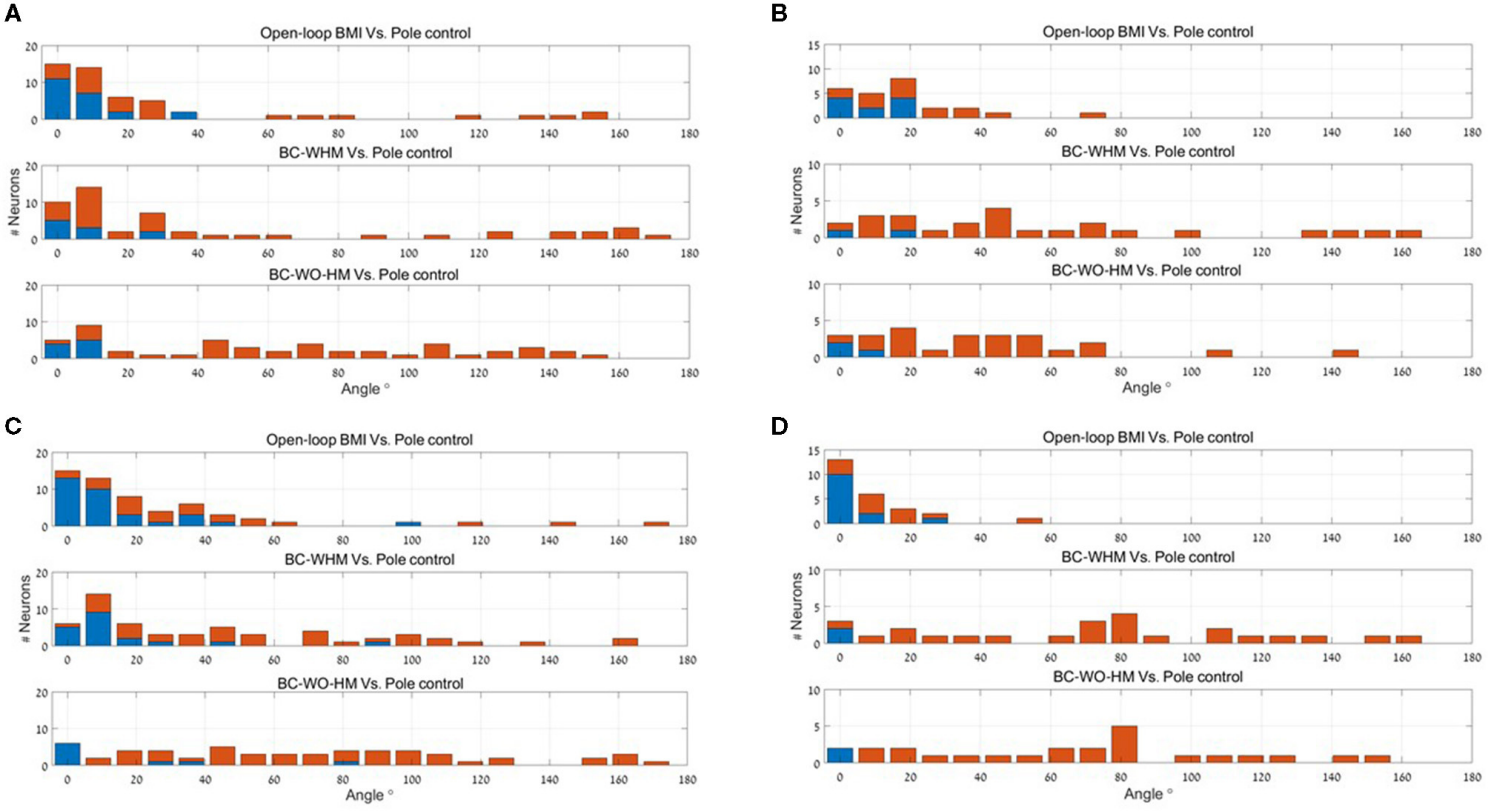
Histograms of the magnitude of EPD shifts at −100 ms lag between different stages of BMI experiments for 56 recorded M1 units **(A)**, 25 simulated M1-like units **(B)**, 55 recorded PMd units **(C)** and 25 simulated PMd-like units **(D)**. Upper panels: magnitude of PD shifts between open-loop BMI and PC. Middle and lower panels: magnitude of EPD shifts between PC and BC-WHM and BC-WO-HM, respectively. Red bars indicate statistically significant shifts (*p* < 0.05).

Figure 3 depicts histograms of the magnitudes (absolute values) of EPD shifts at −100 ms lag, between different stages of BMI experiments for: (a) 56 recorded M1 units, (b) 25 simulated M1-like units, (c) 55 recorded PMd units, and (d) 25 simulated PMd-like units (25). The means and standard deviations of the magnitude of EPD shifts in BMI experiments and in BMI simulations are summarized and compared in Table 1, for M1 units (upper part) and PMd units (lower part) for three lags (0,−100 and −200 ms).

**Table 1:** Mean ± standard deviation of estimated PD shifts (in degrees) between different stages of BMI experiments and simulations.

		**Lag = 0**	**Lag = −100 ms**	**Lag = −200 ms**
**M1**				
**Experiment**				
	PC vs. Open loop BMI	29.1 ± 30.6	30.5 ± 41.7	22.9 ± 31.1
	PC vs. BC-WHM	40.8 ± 39.5	52.8 ± 56.7	37.0 ± 42.3
	PC vs. BC-WO-HM	53.1 ± 39.8	67.6 ± 48.1	76.2 ± 47.2
**Simulation**				
	PC vs. Open loop BMI	25.8 ± 30.0	21.7 ± 17.0	26.2 ± 25.4
	PC vs. BC-WHM	48.7 ± 39.8	44.0 ± 33.7	46.0 ± 35.5
	PC vs. BC-WO-HM	52.2 ± 44.6	60.3 ± 47.2	64.4 ± 48.3
				
	Real vs. PC	35.0 ± 25.7	36.4 ± 27.0	39.6 ± 29.3
**PMd**				
**Experiment**				
	PC vs. Open loop BMI	37.4 ± 36.5	29.8 ± 35.0	38.7 ± 42.7
	PC vs. BC-WHM	39.7 ± 40.0	47.8 ± 42.4	56.6 ± 48.4
	PC vs. BC-WO-HM	53.3 ± 51.2	72.4 ± 47.8	72.4 ± 48.6
**Simulation**				
	PC vs. Open loop BMI	14.4 ± 11.4	12.3 ± 12.9	15.2 ± 15.3
	PC vs. BC-WHM	69.6 ± 42.4	70.1 ± 43.5	69.7 ± 44.8
	PC vs. BC-WO-HM	74.1 ± 47.7	75.2 ± 47.8	75.0 ± 48.0
				
	Real vs. PC	7.0 ± 5.4	8.2 ± 8.0	8.6 ± 9.3

The upper panel of each sub-plot in Figure 3 represent histograms of the magnitude of EPD shifts between PC and open-loop BMI (i.e., shift in PD of the same neural activity with respect to either the velocity predicted by the BMI decoder or pole velocity). Simulations successfully reproduce units with significant EPD shifts from PC to open-loop BMI (44−64% of the recorded or simulated units). The mean EPD shift is similar for simulated M1-like units and recorded M1 units, but is smaller for simulated PMd-like units compared to recorded PMd units. This may indicate that the simulations do not include all the signals that the PMd units encoded during the experiments. In any case the results indicate that the imprecision of the BMI filter contributes to the observed shift in EPD.

The middle and lower panels of each sub-plot in Figure 3 depict histograms of the magnitude of EPD shifts from PC to BC (with and without hand movements, respectively). Table 1 indicates that during BMI experiments, EPD shifts from PC to BC were higher than from PC to open-loop BMI. This phenomenon is successfully reproduced in simulations. Thus, the apparent EPD shifts can be introduced by the BMI filter, even if there is no adaptation or change in context.

### 4.2. PDs in BMI Experiments With Kalman Filters

**Figure 4:**
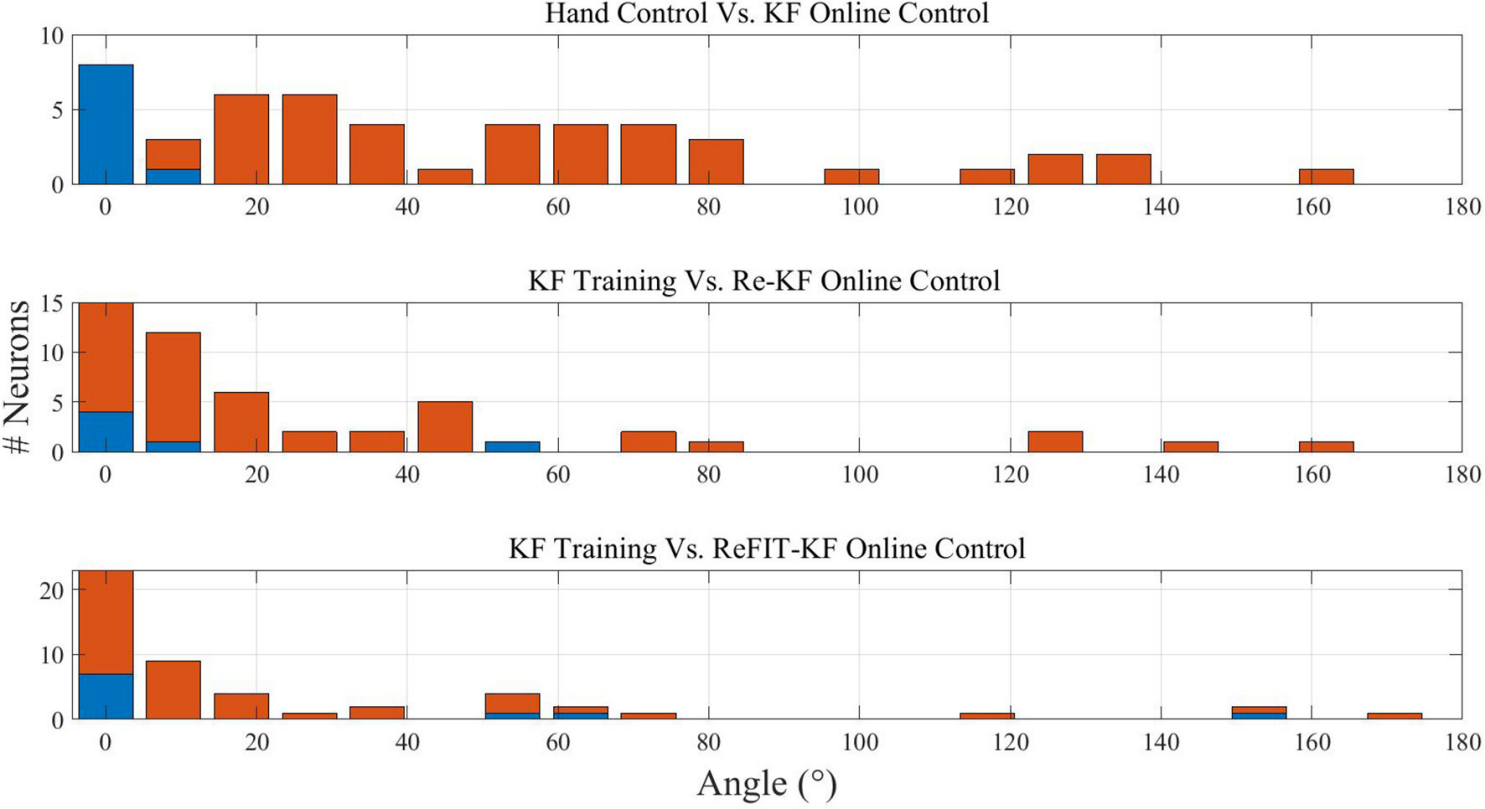
Histograms of the magnitude of EPD shifts at −100 ms lag between stages of simulated BMI experiments with different Kalman filters. Upper: EPD shifts between hand control and KF online control. Middle: EPD shifts between KF training data and Re-KF online control. Lower: EPD shifts between KF training and ReFIT-KF online control. Red bars indicate statistically significant shifts (*p* < 0.05).

**Figure 5:**
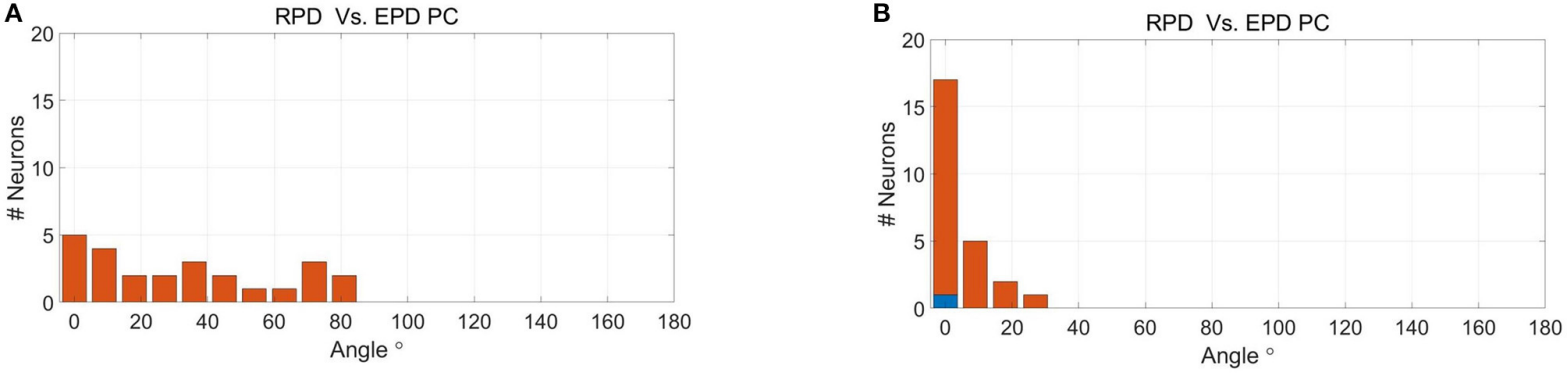
Histogram of magnitude of deviations between RPDs, as defined by the tuning weights of the simulated neural activity with respect to the velocity, and EPD in PC at −100 ms lag for simulated M1-like **(A)** and PMd-like **(B)** units.

BMI experiments with Kalman filters, reported in Fan et al. (2014), investigated PD shifts from hand control to BC with KF (referred to as KF online control) and PD shifts from KF training data, recorded during KF online control, to Re-KF and ReFIT-KF online control. They reveal that (see Figures 4A, 5A and related text in Fan et al., 2014): (1) The distributions of EPD shifts from KF training data to Re-KF or ReFIT-KF online control are narrower than the distribution of EPD shifts from hand control to KF online control, and (2) the distribution of EPD shifts from KF training data to ReFIT-KF online control is narrower than the distribution of EPD shifts from KF training data to Re-KF online control.

Figure 4 demonstrates that those phenomena are successfully reproduced in our simulations. The different panels depict histograms of the magnitude of PD shifts at −100 ms lag between different stages of simulated BMI experiments with KF, re-KF, and reFIT-KF. Note that here we present the distributions of the magnitude (absolute value) of EPD shifts rather than the distributions of the signed EPD shifts (±180 deg). The first three columns of Table 2 summarize the simulation results for three lags: 0, −100 and −200 ms. It is evident that the mean magnitude of EPD shifts between hand control and KF online control is larger than the mean magnitude of EPD shifts between KF training data to Re-KF online control or ReFIT-KF online control.

**Table 2:** Mean ± standard deviation of EPD shifts (in degrees) between stages of simulated BMI experiments with different Kalman filters. EPDs were estimated using either linear regression (Equation 2) on binned data generated in simulations of random movements or using cosine fit of mean neural activity in center-out simulations.

**Movement**	**Random**			**Center-out**
PD estimation		Linear regression		Cosine-fit
Ref		Lebedev et al., 2005		Fan et al., 2014
Shift conditions	Lag = 0	Lag = −100 ms	Lag = −200 ms	
Hand control vs. KF	49.8 ± 37.7	51.6 ± 42.0	53.1 ± 41.7	47.6 ± 40.9
KF training vs. Re-KF	31.0 ± 37.4	33.6 ± 39.0	37.0 ± 40.5	28.5 ± 45.1
KF training vs. ReFIT	28.5 ± 40.3	29.4 ± 42.1	31.4 ± 44.3	21.9 ± 45.9

The above results are based on simulations of movements to random targets and linear regression estimates of PD as in Lebedev et al. (2005). For completeness, the last column of Table 2 indicates that the same phenomena are also apparent when PDs are estimated by fitting a cosine to mean spike counts per direction in center-out simulations, as in Fan et al. (2014). Based on those results, the mean EPD shifts following the transition from KF training to Re-KF or ReFIT-KF decreased by a factor of 1.67 and 2.17, respectively, compared to mean EPD shifts following the initial transition from hand control to KF. Thus, the simulations reproduced not only the PD shifts, but also the effect of re-training (Re-KF) and especially the effect of intention (ReFIT-KF) on narrowing the distribution of PD shifts, though further analysis is needed to compare the magnitude of the effects.

### 4.3. Real vs. Estimated PD

Given that the simulations successfully reproduce the observed shifts in EPD, they provide a powerful tool for further investigation of EPDs. Here we investigate whether EPDs in PC, estimated using Equation 2 with respect to cursor velocity, capture well the RPD with which the unit encodes the estimated velocity, as defined by the corresponding tuning weights and Equation 4. Figure 5 depicts the histogram of the magnitude of deviations between RPD and EPD in PC at −100 ms lag. Table 1 (last column of each part) indicates that the mean magnitude of deviations at 0, −100 and −200 ms range from 35.0–39.6° to 7.0–8.6° for the simulated M1-like and PMd-like units, respectively. These deviations may occur when the conditions of Proposition 3 are not satisfied. In particular, they can be attributed to deviations between RPDs with respect to estimated velocity and RPDs with respect to any other signal that is correlated with velocity (e.g., the control signal). Since only M1-like units encode the control signal, the deviations between EPDs and RPDs are larger for M1-like units compared to PMd-like units.

### 4.4. Filter Effects on EPDs

**Figure 6:**
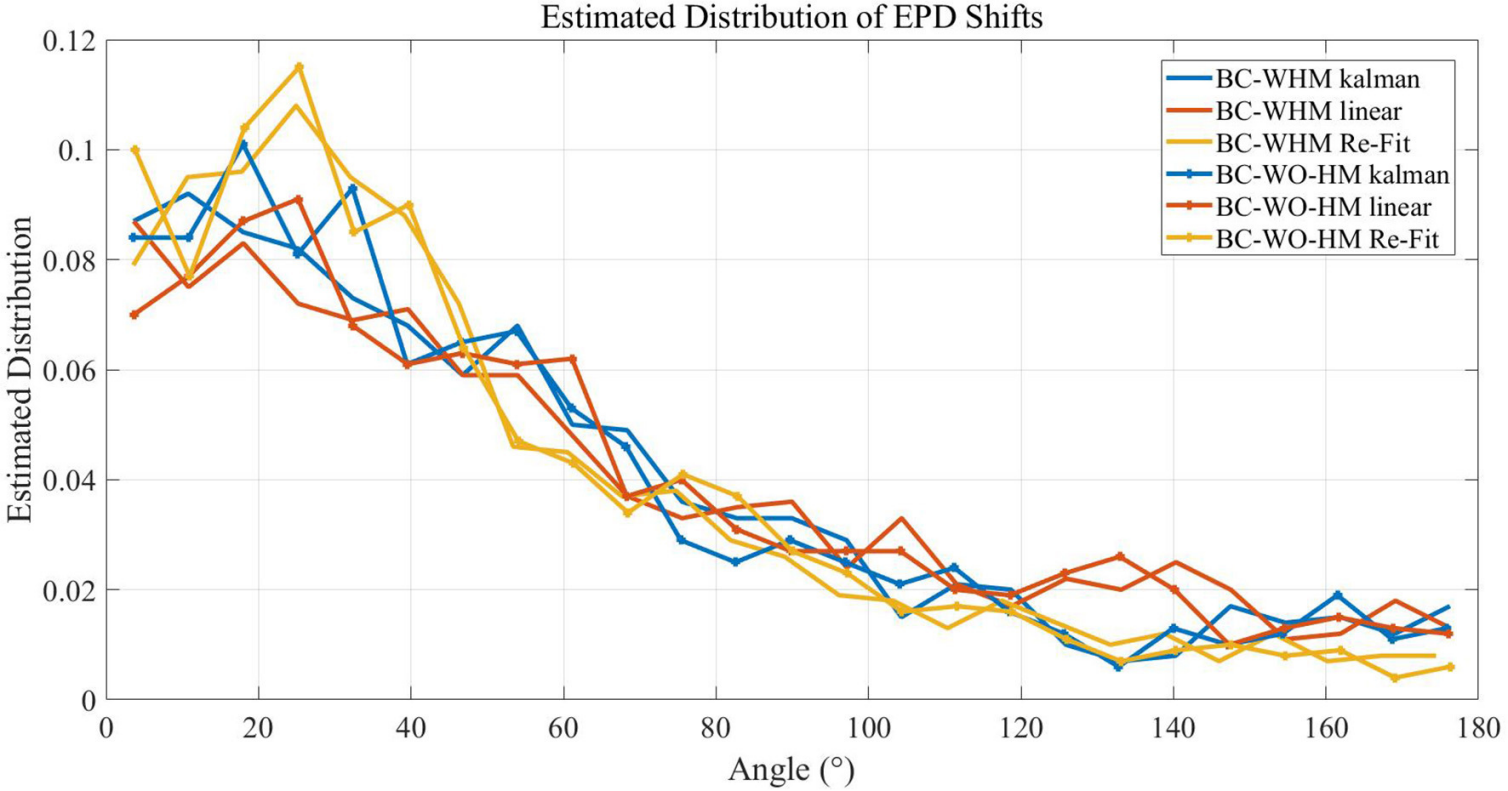
Estimated distributions of the magnitude of EPD shifts between hand control and either BC-WHM or BC-WO-HM during simulations with different filter types (linear, KF, and ReFIT-KF). Distributions were estimated over 20 runs of 20 min with 50 different units each (total of 1,000 simulated units).

Figure 6 compares the distributions of EPD shifts between pole and brain control during simulations with three different BMI filters: linear filter (as in Lebedev et al., 2005), KF and ReFIT-KF (as in Fan et al., 2014). The distributions were estimated from a total of 1,000 simulated units, from 20 runs (of 20min and 50 different units). Statistical analysis indicates that the type of filter has a significant effect on the distribution of EPD shifts in most cases (Wilcoxon signed-rank test, *p* < 0.025, except for the distributions of PD shifts after the transition to BC-WHM with linear compared to KF, *p* = 0.06). However, EPD shifts from hand to brain control are significant in all cases: the mean magnitude of EPD shifts from hand control to BC-WHM is 60.5°, 55.6°, and 49.4° for linear filter, KF and ReFIT-KF, respectively, while the mean magnitude of EPD shifts from hand control to BC-WO-HM is 59.3°, 54.3°, and 48.6°, respectively.

### 4.5. Simulations Supporting Theoretical Analysis

Theoretical conditions under which EPDs in open-loop BMI should remain the same as in PC are specified in Proposition 2, section 3. The first and third conditions require that the components of each of the encoded signals should be uncorrelated across different bins and have the same co-variance. To better satisfy these conditions we simulated center-out BMI experiments to 8 equally spaced directions, and trained the BMI filters on longer sections, (3-times longer: 30 min for training linear and KF, and 60 min for re-calibration). We note that there may still be correlations between the components of the signals, and especially the components of the control signal. The third condition also requires that the tuning weights of the signals that are uncorrelated with the velocity would be ortho-normal, however, this condition is left unsatisfied at this stage. The second condition requires that the regression weights *a*_*PC*_ and *b*_*PC*_ of all the units should be uncorrelated and have the same variance. To better satisfy this condition we increased the number of simulated units from 50 to 150, and assigned them uniformly distributed RPDs.

To better satisfy the conditions of Proposition 2, the effect of signals that are uncorrelated with the velocity was reduced by conducting additional simulations with only PMd-like units, which do not encode the control signal. Furthermore, each PMd-like unit was characterized by a single RPD that was used to encode both the estimated velocity and estimated position. Thus, these simulations also satisfied the conditions for Proposition 3, under which EPDs should equal RPDs.

**Figure 7:**
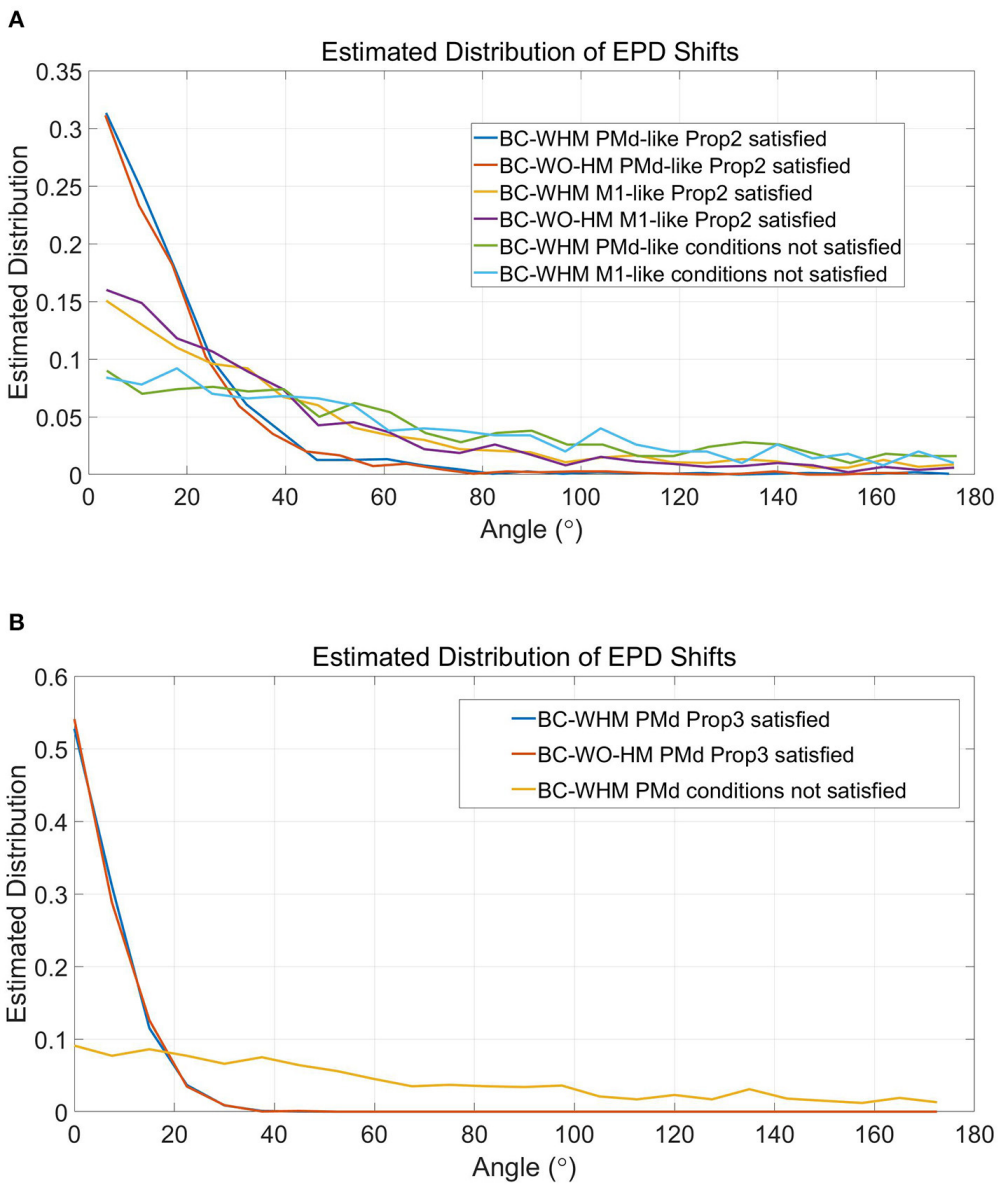
Estimated distributions of the magnitude of EPD shifts during simulations that better satisfy the conditions of Proposition 2 **(A)** and simulations that best satisfy both Propositions **(B)**. In particular, simulations that better satisfy Proposition 2 involved center-out rather than random movements, included 150 units with uniformly distributed RPDs rather than 50 units with random RPDs in each simulation, and used filters that were trained over 3-times longer sections. Simulations that best satisfy both Propositions were similar but included only PMd-like units, each with a single RPD that was used to encode both the estimated velocity and estimated position. Distributions were estimated from 3, 000 simulated units during 20 simulations. Graphs for “conditions not satisfied” are shown for comparison (same as in Figure 6 but shown separately for M1-like and PMd-like units).

Figure 7 depicts estimated distributions of shifts in EPDs during the simulations that better satisfy the conditions of Proposition 2 (Figure 7A), and simulations that best satisfy the conditions of both Propositions (Figure 7B). Since EPDs were estimated using cosine fit, a single PD was estimated for each unit. The distributions were estimated from a total of 3, 000 simulated units from 20 runs (with 150 units each). The distributions for the regular case ('conditions not satisfied') are shown for comparison (same as in Figure 6 but shown separately for M1-like and PMd-like units). The distributions of EPD shifts are clearly more narrow when the conditions of Proposition 2 are better satisfied, especially for PMd-like units which do not encode the control signal. The distributions of EPD shifts become even more narrow when the conditions of both Propositions are best satisfied (Figure 7B), since those simulations involve only PMd-like units. In particular, under the conditions that better satisfy Proposition 2, the mean magnitude of EPD shifts from PC to BC-WHM and BC-WO-HM decreased to 42.8° and 38.2°, respectively, for M1-like units, and to 17.5° and 17.1°, respectively, for PMd-like units. Under the conditions that best satisfy both Propositions, the mean EPD shifts from PC to either BC-WHM or BC-WO-HM decreased further to 8.5° for the single population of PMd-like units. Thus, the simulations support Proposition 2, which lists the conditions for reducing the shifts in EPDs from PC to open-loop BMI.

**Figure 8:**
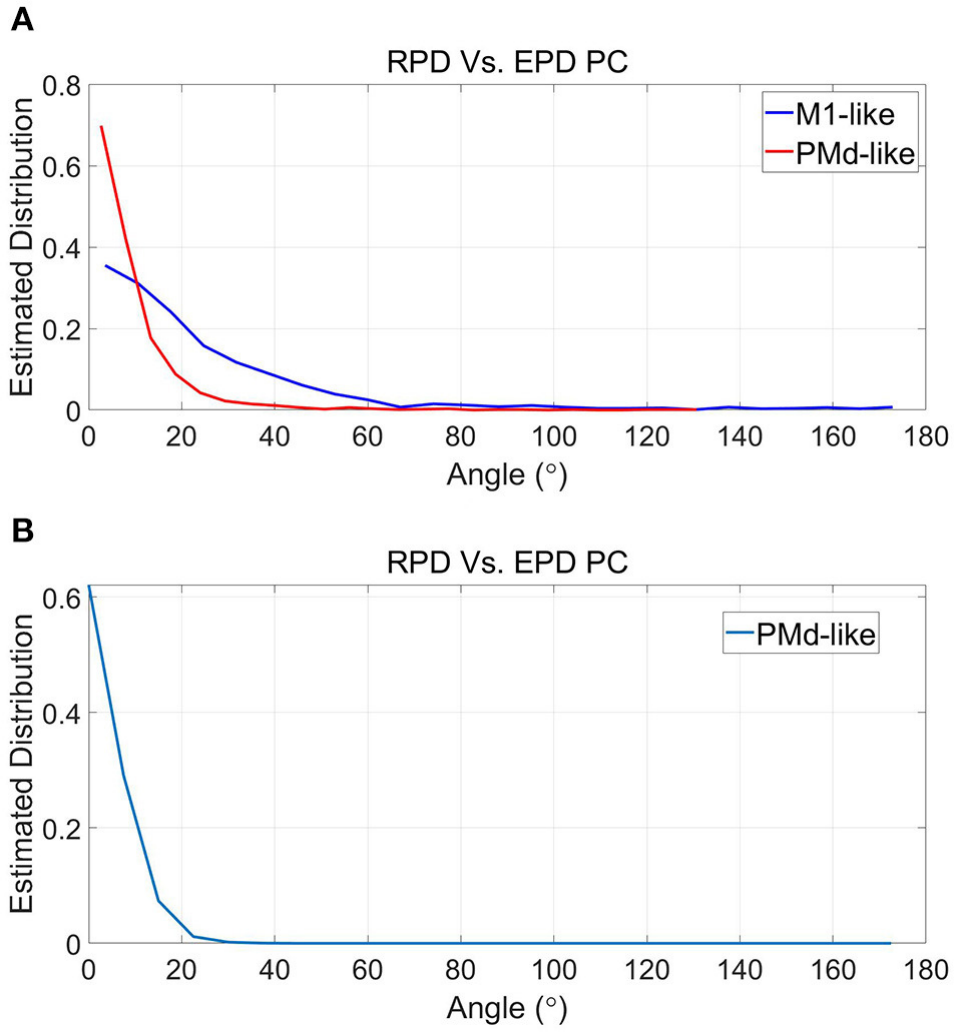
Estimated distributions of the magnitude of deviations between RPD and EPD during simulations that better satisfy the conditions of Proposition 2 **(A)** and simulations that best satisfy both Propositions **(B)**. See Figure 7 and text for more details.

Figure 8 depicts the deviations between RPDs and EPDs in PC for the simulations that better satisfy the conditions of Proposition 2 (Figure 8A) and simulations that best satisfy both Propositions (Figure 8B). Under the conditions that better satisfy Proposition 2, the distribution of the deviations for M1-like units is narrower than before (compare to section 4.3 and Figure 5) with a mean of 24.8°. Nevertheless, it is still wider than the distribution of deviations for PMd-like units, which is similar to that under normal conditions with mean of 9.1°. As mentioned before, the deviations between RPD and EPD are larger for M1-like units since they encode the control signal too. Under the conditions that best satisfy both Propositions, the mean deviations further decreased to 6.9° (for the single populations of PMd-like units). Thus, the simulations supports proposition 3, which lists the conditions under which EPDs should be the same as RPDs.

## 5. Conclusions

We investigated shifts in EPD using simulations in which the brain is assumed to implement state estimation and control, and recorded neurons are assumed to encode the relevant signals in a linear way. Under those assumptions, we demonstrated that the observed shifts in EPDs following the transition to brain control may occur even without any adaptation of the actual tuning weights of the neurons or the internal model. The results captured well the relative magnitude of the EPD shifts reported in the literature, with different types of BMI filters. Thus, we conclude that the observed EPD shifts after the transition to brain control or after switching to a new BMI filter or stage, may not imply neural adaptation.

Focusing on BMI experiments with linear filters, we demonstrated that part of the shift in EPDs is already evident when comparing EPDs with respect to hand velocity (pole control) with EPDs with respect to the velocity predicted by the BMI filter (open-loop BMI). Theoretical analysis, supported by simulation results, reveals the conditions under which these shifts could be minimized. Some conditions are under experimental control, and in particular the lack of correlation between the components of velocity during BMI training. However, other conditions, and in particular the lack of correlation between the components of the tuning weights, are not. We demonstrated that simulations that better satisfy those conditions result in significantly smaller EPD shifts from hand to brain control. We conclude that under the assumptions of our model, the observed EPD shifts may result from imperfect BMI filters, due to experimental conditions, and in particular correlated velocities or tuning weights.

The theoretical relationship between the estimated tuning weights in open-loop BMI (α_*BMI*_) and in PC (α_*PC*_, Equation 9), depends on the co-variance of the encoded signals that are uncorrelated with the velocity (third condition in Corollary to Proposition 2). Since those signals and the corresponding tuning weights are unknown, this co-variance matrix cannot be computed. Thus, the analysis provides a basis for evaluating the conditions for minimizing the shifts and the effect on the distribution of EPDs rather than predicting EPD shifts for specific units.

Focusing on BMI experiments with Kalman filters, before and after re-calibration, the simulations reproduce the observed EPD shifts and the effect of re-calibration on their magnitude Fan et al. (2014). In particular, (1) the magnitudes of EPD shifts between KF training data and Re-KF or ReFIT-KF online control were smaller than those between hand control and KF online control. (2) the magnitude of EPD shifts between KF training data and ReFIT-KF were smaller than those between KF training data and Re-KF. Thus, the effect of re-calibration of KF on PD shifts is reproduced well even with no adaptation.

The simulations facilitate further investigations into EPD shifts that are impossible to perform in actual experiments. In particular, we demonstrated that when neurons encode the estimated velocity, estimated position and control signal with different RPDs (i.e., the RPD of the estimated velocity differs from the RPD for estimated position or control vector), the EPD with respect to actual velocity may not capture the RPD at which the neuron encode the estimated velocity.

Our simulation is based on optimal state estimation and control, which was proposed as a viable model for motor control during reaching movements (Todorov and Jordan, 2002; Todorov, 2005; Shadmehr and Krakauer, 2008). We have previously developed this simulation for investigating BMI experiments and demonstrated that it successfully explains the observed changes in neural modulations (Benyamini and Zacksenhouse, 2015). Here we demonstrated that the same simulation successfully reproduces the observed shifts in EPD (Carmena et al., 2003) following the transition to brain control without any adaptation of the actual tuning weights of the neurons or the internal model. Furthermore, the simulation was extended to include also brain control with different Kalman filters as in Fan et al. (2014), and successfully reproduced the observed EPD shifts with those filters too.

Adaptation processes may still occur, but are not necessary to explain the observed shifts in EPDs after the transition to brain control or after switching to a new BMI filter or stage. However, long term adaptation may be required to explain the observed shifts in EPDs while using the same BMI filter (Ganguly and Carmena, 2009). Further analysis is needed to investigate whether those changes necessarily imply changes in the actual tuning properties of the units or can be explained by adapting only the internal model. Internal model adaptation refers to changing the internal representation of the dynamics of the cursor to reflect its behavior in brain control, when it depends on the BMI filter, rather than in pole control, when it follows the muscle activated hand.

Our work indicates that EPD shifts are smaller when the tuning weights of the units used in the BMI filter are uncorrelated, so it might be advantageous to use a subset of units that are more uniformly distributed. However, further analysis is needed to investigate the trade-off between the number of units used for the BMI filter and the uniformity of the distribution of their PDs.

In summary, our work questions the common assumption that observed EPD shifts after the transition to brain control reflect neural adaptation. Instead, we conclude that the observed EPD shifts may result from imperfect BMI filters, due to experimental conditions, and in particular correlated velocities, correlations between components of other encoded signals or non-uniformly distributed RPDs among the decoded units. Our investigation provides theoretical and simulation tools for better understanding RPD shifts and BMI experiments.

## Data Availability Statement

The data analyzed in this study is subject to the following licenses/restrictions: privately obtained from other Labs. Requests to access these datasets should be directed to Nicolelis' Lab, nicoleli@neuro.duke.edu.

## Author Contributions

MB and MZ contributed to conception and design of the study, contributed to manuscript revision, read, and approved the submitted version. MB built the simulation, conducted simulations, analyzed experimental and simulations results, performed theoretical analysis, and wrote the first version of the manuscript. Both authors contributed to the article and approved the submitted version.

## Conflict of Interest

The authors declare that the research was conducted in the absence of any commercial or financial relationships that could be construed as a potential conflict of interest.
