# Supplementary material for: Shifts in Estimated Preferred Directions During Simulated BMI Experiments With No Adaptation
Source: Front Syst Neurosci. 2021 Jul 19;15:677688. doi: 10.3389/fnsys.2021.677688 (PMC8326909; doi:10.3389/fnsys.2021.677688)
Supplement: Supplementary file 1 [file Data_Sheet_1.pdf]

Shifts in Preferred Direction During Simulated BMI Experiments With No Adaptation: Supplementary Material

1 PROPOSITION 1 AND ITS PROOF

1.1 Preliminaries

Theoretical analysis is based on the assumption that the neural activity encodes the velocity, V, and other signals that are either correlated or uncorrelated with the velocity (denoted by C & U, respectively). Thus, the mean subtracted spike counts $N \in \mathbb{R}^{N_n \times T_n}$ of N_n neurons in T_n bins is related to the mean subtracted $V = [V_x\ V_y] \in \mathbb{R}^{2 \times T_n}$, signals $C \in \mathbb{R}^{d_C \times T_n}$ and $U \in \mathbb{R}^{d_U \times T_n}$, by:

$$N = W_V V + W_C C + W_U U + \epsilon \qquad \text{(S1)}$$

where d_C and d_U are the dimensions of the correlated and uncorrelated signals, respectively, $W_V \in \mathbb{R}^{N_n \times 2}$, $W_C \in \mathbb{R}^{N_n \times d_C}$, $W_U \in \mathbb{R}^{N_n \times d_U}$ are the tuning weights of V, C and U respectively, and ϵ is the neural noise, which is assumed to be uncorrelated with any of the other signals.

To facilitate theoretical analysis, it is further assumed that the correlated signals can be expressed as $C = R_V V + R_Z Z$ where Z is uncorrelated with V, so:

$$N = (W_V + W_C R_V)V + S \qquad \text{(S2)}$$

where $S = W_C R_Z Z + W_U U + \epsilon$.

The velocity tuning weights can be estimated by linear regression:

$$\alpha = N V^{+} = N V^T (V V^T)^{-1} \in \mathbb{R}^{N_n \times 2} \qquad \text{(S3)}$$

The estimated tuning weights in PC, α_{PC}, and in open-loop BMI, α_{BMI}, are computed by regressing the neural activity in PC on either the hand velocity or the velocity predicted by the BMI filter, respectively.

1.2 Proposition 1A and its proof

Proposition 1A: Assuming the neural activity can be modeled as in Eq S2, the tuning weights in pole control, α_{PC}, are given by:

$$\alpha_{PC} \approx W_V + W_C R_V \qquad \text{(S4)}$$

Proof of Proposition 1A: Since S is uncorrelated with V, $SV^T \approx 0$. Thus, Eq. S4 can be derived directly by inserting Eq. S2 in Eq. S3:

$$\alpha_{PC} = ((W_V + W_C R_V)V + S)V^T (V V^T)^{-1} \approx (W_V + W_c R_V). \qquad \text{(S5)}$$

1.3 Proposition 1B and its proof

Proposition 1B relates the tuning weights in open-loop BMI to tuning weights in PC for the simple case when the BMI filter depends only on the current binned spike counts. Proposition 1C extends this to the general case when the BMI filter depends on the spike counts in the current and $L-1$ bins.

Proposition 1B: Assuming the neural activity can be modeled as in Eq S2, and the BMI filter is based only on the current binned neural activity then the tuning weights in open-loop BMI, α_{BMI}, are given by:

$$\alpha_{BMI} \approx \alpha_{PC} + \Sigma_S \alpha_{PC} (\Sigma_V \Sigma_{\alpha_{PC}})^{-1} / N_n \tag{S6}$$

Where $\Sigma_S = SS^T / T_n, \Sigma_V = VV^T / T_n$ and $\Sigma_{\alpha_{PC}} = \alpha_{PC}^T \alpha_{PC} / N_n$ are the co-variance matrices of S, V and α_{PC}, respectively.

Proof of Proposition 1B: In the simple case, the predicted velocity $\hat{V} \in \mathbb{R}^{2 \times T_n}$ is a linear function of the zero mean neural activity $N \in \mathbb{R}^{N_n \times T_n}$:

$$\hat{V} = W_{BMI} N \tag{S7}$$

Where $W_{BMI} \in \mathbb{R}^{2 \times N_n}$ is the BMI decoder weights.

In the simple case, the weights of the linear BMI filter are determined by linear regression of the zero-mean velocity V_t on the zero-mean neural activity N_t recorded for training:

$$W_{BMI} = V_t N_t^+ = V_t N_t^T \{N_t N_t^T\}^{-1} = \frac{1}{T_n} V_t N_t^T \Sigma_N^{-1} \tag{S8}$$

Where $\Sigma_N = \frac{1}{T_n} N_t N_t^T$ is the co-variance matrix of the neural activity estimated from the training neural activity.

Open loop BMI tuning weights are computed by inserting Eq.S7 in Eq. S3:

$$\begin{aligned}
&\alpha_{BMI} = N(W_{BMI}N)^T \{(W_{BMI}N)(W_{BMI}N)^T\}^{-1} \qquad \text{(S9)}\\
&= NN^T W_{BMI}^T \{W_{BMI} NN^T W_{BMI}^T\}^{-1}\\
&= NN^T \frac{1}{T_n} \Sigma_N^{-1} N_t V_t^T \{\frac{1}{T_n} V_t N_t^T \Sigma_N^{-1} T_n \Sigma_N \frac{1}{T_n} \Sigma_N^{-1} N_t V_t^T\}^{-1}\\
&= \Sigma_N \Sigma_N^{-1} N_t V_t^T \{\frac{1}{T_n} V_t N_t^T \Sigma_N^{-1} N_t V_t^T\}^{-1}\\
&= T_n N_t V_t^T \{V_t N_t^T \Sigma_N^{-1} N_t V_t^T\}^{-1}\\
&\Rightarrow \alpha_{BMI} V_t N_t^T \Sigma_N^{-1} N_t V_t^T = T_n N_t V_t^T\\
&\Rightarrow \alpha_{BMI} V_t N_t^T = T_n \Sigma_N
\end{aligned}$$

Under the assumption that the neural activity encodes the velocity and other signals (S) as defined by Eq. S2, and that those signals are uncorrelated with the velocity, V, the co-variance matrix of the neural activity is:

$$\Sigma_N \approx (W_V + W_c R_V) \Sigma_V (W_V + W_c R_V)^T + \Sigma_S \approx \alpha_{PC} \Sigma_V \alpha_{PC}^T \tag{S10}$$

where the last approximation is based on Eq. S4.

Inserting Eq. S2 in Eq. S9, and using Eq. S10, the open-loop BMI tuning weights are:

$$\alpha_{BMI} V_t N_t^T = \alpha_{BMI} V_t ((W_V + W_c R_V) V_t + S)_n^T \approx T_n \alpha_{BMI} \Sigma_V (W_V + W_c R_V)^T = T_n \Sigma_N \tag{S11}$$
$$\alpha_{BMI} \Sigma_V (W_V + W_c R_V)^T = (W_V + W_c R_V) \Sigma_V (W_V + W_c R_V)^T + \Sigma_S$$
$$\Rightarrow \alpha_{BMI} = (W_V + W_c R_V) + \Sigma_S \{\Sigma_V (W_V + W_c R_V)^T)\}^+$$

Using Eq. S4, the last expression can be simplified to:

$$\alpha_{BMI} = \alpha_{PC} + \Sigma_S \{\Sigma_V \alpha_{PC}^T)\}^+ \tag{S12}$$

Opening the pseudo-inverse, and denoting $\Sigma_{\alpha_{PC}} = \alpha_{PC} \alpha_{PC}^T / N_n$, Eq. S12 leads to Eq. S13.

1.4 Proposition 1C and its proof

Proposition 1C: Assuming the neural activity can be modeled as in Eq S2, and that the BMI filter includes multiple lags of bin-width B_W, the tuning weights in open-loop BMI, $\alpha_{BMI}(j)$, in lag $\tau = jB_W$, are given by:

$$\alpha_{BMI}(j) = \alpha_{PC} \Sigma_V (|j - i|) \Sigma_V^{-1}(i) + \Sigma_S (|j - i|) \alpha_{PC} (\Sigma_V (i) \Sigma_{\alpha_{PC}})^{-1} / N_n \tag{S13}$$

where $\Sigma_S(j) = S_k S_{k-j}^T / T_n$ and $\Sigma_V(j) = V_k V_{k-j}^T / T_n$ are the co-variance matrices of S and V, respectively, at lag $\tau = jB_W$, and $\Sigma_{\alpha_{PC}} = \alpha_{PC}^T \alpha_{PC} / N_n$ is the co-variance matrix of α_{PC}.

Proof of Proposition 1C: Considering the multi-lag case, we first arrange the zero-mean neural activity in L lags as:

$$\tilde{N} = \begin{bmatrix} N_k \\ N_{k-1} \\ \cdot \\ \cdot \\ \cdot \\ N_{k-L} \end{bmatrix} = \begin{bmatrix} \alpha_{PC} V_k + S_k \\ \alpha_{PC} V_{k-1} + S_{k-1} \\ \cdot \\ \cdot \\ \cdot \\ \alpha_{PC} V_{k-L} + S_{k-L} \end{bmatrix} \tag{S14}$$

where the last approximation is based on Eq. Eq. S2 and Eq. S4.

The predicted velocity $\hat{V} \in \mathbb{R}^{2 \times T_n}$ is a linear function of $\tilde{N} \in \mathbb{R}^{(N_n \cdot L) \times T_n}$:

$$\hat{V} = W_{BMI} \tilde{N} \tag{S15}$$

where $W_{BMI} \in R^{2 \times (N_n \cdot L)}$ is the BMI decoder weights. Denote the co-variance matrix of the neural activity with lags by $\Sigma_{\tilde{N}} = \frac{1}{T_n} \tilde{N}_k \tilde{N}_k^T$:

$$\Sigma_{\tilde{N}} = \frac{1}{T_n} \tilde{N} \tilde{N}^T = \begin{bmatrix} \Sigma_N(0) & \Sigma_N(1) & \cdot & \cdot & \Sigma_N(L) \\ \Sigma_N(1) & \Sigma_N(0) & \cdot & \cdot & \Sigma_N(L-1) \\ \cdot & \cdot & \cdot & \cdot & \cdot \\ \cdot & \cdot & \cdot & \cdot & \cdot \\ \Sigma_N(L) & \Sigma_N(L-1) & \cdot & \cdot & \Sigma_N(0) \end{bmatrix} \tag{S16}$$

and

$$\Sigma_N(j) = \frac{1}{T_n} (\alpha_{PC} V_k + S_k)(\alpha_{PC} V_{k-j} + S_{k-j})^T = \alpha_{PC} \Sigma_V(j) \alpha_{PC}^T + \Sigma_S(j)$$

The weights of the decoder are determined by linear regression of the neural activity with lags $\tilde{N}_t$ and velocity V_t recorded for training:

$$W_{BMI} = V_t \tilde{N}_t^{+} = V_t \tilde{N}_t^T \{\tilde{N}_t \tilde{N}_t^T\}^{-1} = \frac{1}{T_n} V_t \tilde{N}_t^T \Sigma_{\tilde{N}}^{-1} \tag{S17}$$

The tuning weights of the open-loop BMI at lag $\tau = jB_W$ are calculated from Eq. S3 using Eq. S15:

$$\begin{aligned}
&\alpha_{BMI}(j) = N_{k-j}\hat{V}_k^{+} = N_{k-j}\hat{V}_k^T(\hat{V}_k\hat{V}_k^T)^{-1} \\
&= N_{k-j}(W_{BMI}\tilde{N}_k)^T(W_{BMI}\tilde{N}_k\tilde{N}_k^T W_{BMI}^T)^{-1} \\
&= N_{k-j}\tilde{N}_k^T W_{BMI}^T(W_{BMI}T\Sigma_{\tilde{N}}W_{BMI}^T)^{-1} \\
&= \begin{bmatrix}\Sigma_N(j) & \Sigma_N(j-1) & \cdot & \cdot & \Sigma_N(j-L)\end{bmatrix} W_{BMI}^T(W_{BMI}\Sigma_{\tilde{N}}W_{BMI}^T)^{-1}
\end{aligned} \tag{S18}$$

Arranging the tuning weights for L lags in $\tilde{\alpha}_{BMI}$:

$$\tilde{\alpha}_{BMI} = \begin{bmatrix}\alpha_{BMI}(0) \\ \alpha_{BMI}(1) \\ \cdot \\ \cdot \\ \alpha_{BMI}(L)\end{bmatrix} \tag{S19}$$

and inserting Eq.S17 we get:

$$\begin{aligned}
&\tilde{\alpha}_{BMI} = \Sigma_{\tilde{N}}W_{BMI}^T(W_{BMI}\Sigma_{\tilde{N}}W_{BMI}^T)^{-1} \\
&= \Sigma_{\tilde{N}}(\frac{1}{T_n}V_k\tilde{N}_k^T\Sigma_{\tilde{N}}^{-1})^T((\frac{1}{T_n}V_k\tilde{N}_k^T\Sigma_{\tilde{N}}^{-1})\Sigma_{\tilde{N}}(\frac{1}{T_n}V_k\tilde{N}_k^T\Sigma_{\tilde{N}}^{-1})^T)^{-1} \\
&= T_n\tilde{N}_kV_k^T(V_k\tilde{N}_k^T\Sigma_{\tilde{N}}^{-1}\tilde{N}_kV_k^T)^{-1} \\
&\Rightarrow \tilde{\alpha}_{BMI}V_k\tilde{N}_k^T\Sigma_{\tilde{N}}^{-1}\tilde{N}_kV_k^T = T_n\tilde{N}_kV_k^T \\
&\Rightarrow \tilde{\alpha}_{BMI}V_k\tilde{N}_k^T = T_n\Sigma_{\tilde{N}}
\end{aligned} \tag{S20}$$

Expressing Eq. S20 explicitly:

$$\begin{bmatrix}\alpha_{BMI}(0) \\ \alpha_{BMI}(1) \\ \cdot \\ \cdot \\ \alpha_{BMI}(L)\end{bmatrix} V_k \begin{bmatrix}N_k^T & N_{k-1}^T & \cdot & \cdot & \cdot & N_{k-L}^T\end{bmatrix} = T \begin{bmatrix}\Sigma_N(0) & \Sigma_N(1) & \cdot & \cdot & \Sigma_N(L) \\ \Sigma_N(1) & \Sigma_N(0) & \cdot & \cdot & \Sigma_N(L-1) \\ \cdot & \cdot & \cdot & \cdot & \cdot \\ \cdot & \cdot & \cdot & \cdot & \cdot \\ \Sigma_N(L) & \Sigma_N(L-1) & \cdot & \cdot & \Sigma_N(0)\end{bmatrix} \tag{S21}$$

Inserting Eq. S2:

$$\begin{bmatrix} \alpha_{BMI}(0) \\ \alpha_{BMI}(1) \\ \cdot \\ \cdot \\ \alpha_{BMI}(L) \end{bmatrix} V_k \begin{bmatrix} V_k^T \alpha_{PC}^T + S_k^T & V_{k-1}^T \alpha_{PC}^T + S_{k-1}^T & . & . & . & V_{k-L}^T \alpha_{PC}^T + S_{k-L}^T \end{bmatrix} = T\Sigma_{\tilde{N}} \tag{S22}$$

Recalling that $\Sigma_V(j) = V_k V_{k-j}^T / T_n$:

$$\begin{bmatrix} \alpha_{BMI}(0) \\ \alpha_{BMI}(1) \\ \cdot \\ \cdot \\ \alpha_{BMI}(L) \end{bmatrix} T_n \begin{bmatrix} \Sigma_V(0)\alpha_{PC}^T & \Sigma_V(1)\alpha_{PC}^T & . & . & . & \Sigma_V(L)\alpha_{PC}^T \end{bmatrix} = T_n \Sigma_{\tilde{N}} \tag{S23}$$

Equating the elements on the jth row and $i^{th}-$ column on both sides:

$$\alpha_{BMI}(j)\Sigma_V(i)\alpha_{PC}^T = \Sigma_N(|j-i|) = \alpha_{PC}\Sigma_V(|j-i|)\alpha_{PC}^T + \Sigma_S(|j-i|) \tag{S24}$$

Thus:

$$\begin{aligned} \alpha_{BMI}(j) &= \alpha_{PC}\Sigma_V(|j-i|)\alpha_{PC}^T(\Sigma_V(i)\alpha_{PC}^T)^+ + \Sigma_S(|j-i|)(\Sigma_V(i)\alpha_{PC}^T)^+ \\ &= \alpha_{PC}\Sigma_V(|j-i|)\Sigma_V(i)^{-1} + \Sigma_S(|j-i|)\alpha_{PC}(\Sigma_V(i)\Sigma_{\alpha_{PC}})^{-1}/N_n \end{aligned} \tag{S25}$$

where $\Sigma_{\alpha_{PC}} = \alpha_{PC}^T \alpha_{PC} / N_n$. This concludes the Proof of Proposition 1C.
